# An Uncommon Case of Sinonasal Adenoid Cystic Carcinoma Metastatic to the Kidney Treated with Metastasectomy

**DOI:** 10.15586/jkcvhl.v11i3.306

**Published:** 2024-08-30

**Authors:** Alyssa M. Lombardo, Tyler Sheetz, Ricardo L. Carrau, Debra L. Zynger, Eric A. Singer

**Affiliations:** 1 Division of Urologic Oncology, The Ohio State University Comprehensive Cancer Center, Columbus, OH;; 2 Department of Otolaryngology, The Ohio State University Comprehensive Cancer Center, Columbus, OH;; 3 Department of Pathology, The Ohio State University Wexner Medical Center, Columbus, OH

**Keywords:** Adenoid cystic carcinoma, Metastasectomy, Metastatic, Nephrectomy, Urologic oncology

## Abstract

Adenoid cystic carcinoma (ACC) is a rare tumor, accounting for 1% of all head and neck cancers, with an aggressive nature characterized by local recurrence, delayed metastasis, and survival of less than 50% at 10 years. This is a case of biopsy-proven ACC to the kidney, 1 of 29 known occurrences, managed by metastasectomy by robotic-assisted nephrectomy, with plans for resection of lung metastasis. Thirteen years after diagnosis of sinonasal ACC treated with resection, the patient presented with shortness of breath. This prompted a CT scan of the chest, which led to the incidental finding of left renal mass and pulmonary lesion. Literature suggests improved disease-specific survival in locoregional recurrence treated with surgery versus radiation; in patients with metastasis to the lung, metastasectomy offers greater survival benefit than supportive therapy. But, this is not significantly better than chemotherapy or radiation alone. While the optimal therapeutic approach remains to be identified in distant metastatic ACC, metastasectomy remains a viable option for patients who have potentially completely resectable metastatic tumors, appropriate performance status, and adequate affected-organ function. Preoperative counseling should include discussion on partial nephrectomy with prioritization of nephron-sparing but potential for increased perioperative risk versus radical nephrectomy to ensure negative margins and expedite timeline to systemic therapy.

## Introduction

Adenoid cystic carcinoma (ACC) is a rare tumor, accounting for only 1% of all head and neck cancers and 10% of all epithelial tumors of the salivary gland (1, 2). It is typically found in the major salivary glands but other affected sites include the minor salivary glands, sinonasal tract, trachea, esophagus, breast, and lung (2, 3). ACC typically presents in the fourth to sixth decade of life with slight female predominance (3:^2^) (2). Despite its slow-growing nature and relatively asymptomatic course, it is an aggressive tumor. It is characterized by high rate of neural invasion, local recurrences, and delayed metastasis. ACC is associated with poor outcomes with a 10-year survival of less than 50%, regardless of the histological grade (4). Common sites of metastasis include lungs, bone, liver, and brain. ACC metastasis to the kidney is extremely rare, with 29 cases reported in the English-language literature, including 9 cases of bilateral kidney metastasis (1, 5). We present the case of a 62-year-old female with biopsy-proven ACC metastatic to the kidney, managed by robotic-assisted radical nephrectomy (metastasectomy) and planned adjuvant systemic therapy, with special attention paid to the perspective and knowledge required for the urologist. This case report was reviewed by the Institutional Privacy Office, and patient consent was obtained for this publication.

## Case Report

This is the case of a 62-year-old female with a history of pathologic T3 ACC of the right maxillary sinus status-post right partial maxillectomy and right nasopharyngectomy with local microscopic disease recurrence 4 years later in the trigeminal (V2) and vidian nerves status-post endoscopic endonasal dissection of vidian and V2 nerve bundle, resection of the pterygomaxillary/sphenopalatine fossa, middle cranial fossa tumor, and maxillary and median nerves, at which point she demonstrated no evidence of disease.

**Figure 1:**
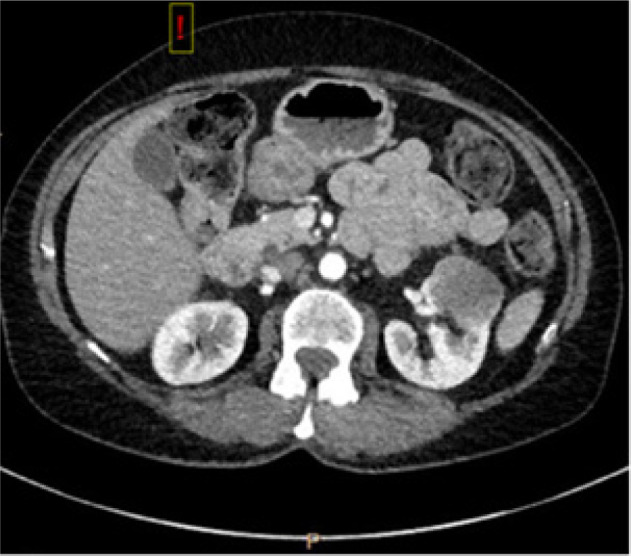
CT scan ordered by urgent care for presentation of shortness of breath with incidental finding of partially imaged heterogeneous, solid-appearing mass in the left kidney, approximately 3.4 × 2.6 cm.

**Figure 2:**
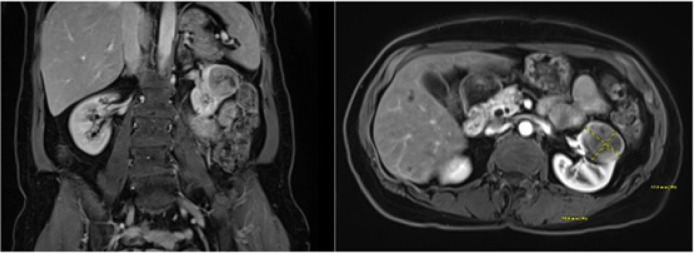
MRI of the abdomen with T1-weighted images in coronal and axial views.

**Figure 3:**
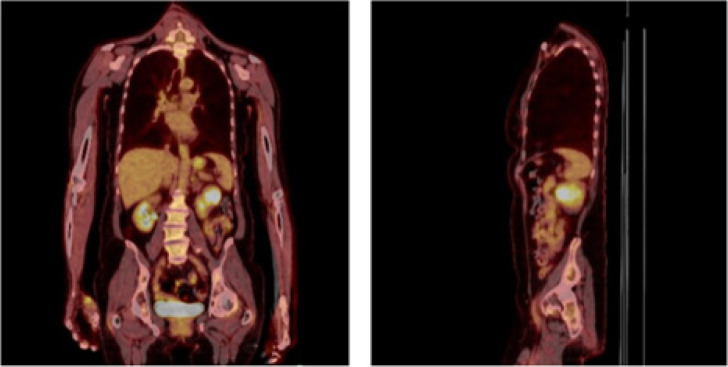
PET scan which demonstrated hypermetabolic left renal mass and axillary lymph node (not pictured).

Nine years later, the patient presented to an urgent care with upper respiratory symptoms, including cough, congestion, sore throat, and shortness of breath, for a duration of three 3 days that were refractory to over-the-counter medications. A chest x-ray was negative for airspace disease, but given the history and concern for metastatic disease, CT of the chest was ordered. The patient followed-up with the medical oncology team for review of imaging, which revealed an interval increase in the size of a spiculated lung lesion seen on a CT scan from 2 years prior, enlarged left axillary lymph node, and a new, partially imaged, heterogeneous solid-appearing mass in the anterior cortex of the mid- to upper pole of the left kidney approximately 3.4 × 2.6 cm in size (Figure 1). At that time, MRI of the abdomen (Figure 2) was performed for better characterization of the mass, and PET scan was done which demonstrated hypermetabolic activity in the areas of reference on the CT scan (Figure 3). Referral to interventional radiology was placed for ultrasound-guided biopsies, and left renal mass biopsy was performed. A referral to cardiothoracic surgery was placed for biopsy of an upper lobe pleural nodule, and perihilar cluster of nodules were seen on imaging that were not amendable to ultrasound-guided biopsy. The renal biopsy showed metastatic ACC. Immunostains for CK7, CD117, p40, and SMA were positive, and calponin and PAX8 were negative.

**Figure 4:**
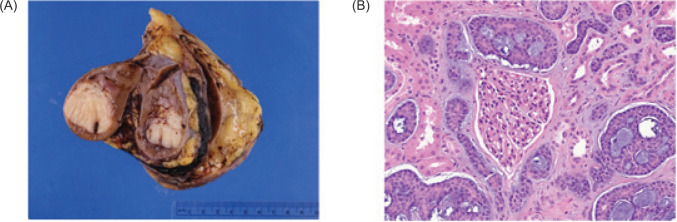
Pathologic findings of adenoid cystic carcinoma (ACC) metastatic to the kidney. (A) Left nephrectomy specimen with a 3.8 cm unifocal, nodular, homogenous, and cream-colored tumor involving the renal parenchyma and renal sinus adipose. (B) Photomicrograph showing a cribriform and tubular tumor with basophilic material within luminal spaces, characteristic of ACC. Residual renal parenchyma is present (glomerulus in center; renal tubules interspersed).

Based on the nature of the patient’s disease, the otolaryngologic oncology team recommended consolidation of tumor burden with emphasis on negative margins, which was communicated to the urologic oncologist via referral. The patient established care with urologic oncology to discuss metastasectomy, which could be accomplished via robotic surgery. Soon thereafter, the patient underwent surgery for which robotic-assisted partial nephrectomy was planned. Intraoperative ultrasound raised concern for tumor invasion into the renal sinus, so a robotic-assisted total nephrectomy was completed to ensure negative margins. She did not experience any complications in the immediate postoperative period and was discharged on postoperative day 2. Gross examination of the specimen noted a 3.8 × 3.6 × 3.0 cm, indurated, vaguely waxy, homogeneous, pale tan-yellow mass at the mid lower pole (Figure 4A). Pathologic diagnosis was consistent with metastatic ACC (Figure 4B). There were single foci of pelvicalyceal urothelial involvement. Surgical margins were negative.

When this patient was seen at urologic oncology follow-up at 2 weeks postoperatively, she was doing well and recovering as anticipated. At the most recent urologic oncology visit 1 year after surgery, the patient’s creatinine is 0.8 from 0.6 preoperatively, and there are plans for abdominal CT at 18 months postoperatively. The patient was also initially referred to cardiothoracic surgery for the evaluation of pleural nodule and perihilar nodularity concerning for metastatic disease. Evaluation by cardiothoracic surgery was that the perihilar nodularity may be related to infection given the patient’s recent presentation with upper respiratory symptoms at the time of renal mass diagnosis. Video-assisted thorascopic surgery (VATS) pleural wedge resection of the hypermetabolic peripheral pleural nodule was recommended. Initially, the patient preferred to avoid surgical intervention and planned for repeat CT imaging of the chest every 3 months. At the most recent cardiothoracic surgery follow-up appointment, 1 year after metastasectomy, CT imaging of the chest demonstrated that the lung nodule was relatively stable in size but was significantly larger than when it was first noted 15 months prior; the patient had decided to pursue preoperative workup for VATS. At the initial medical oncology visit, systemic therapy was deferred to first address possible VATS resection of pleural lesion, and there were plans to revisit systemic treatment in the future. At the most recent medical oncology visit, the patient reported eye pain, and an MRI scan was ordered to investigate for local recurrence. At the most recent follow-up visit at 1 year post-metastasectomy, the patient continues to be under the care of a multidisciplinary team, including medical oncology, urologic oncology, otolaryngology, and cardiothoracic surgery. This case was listed in a prior case series publication, with no radiologic or surgical details included in that report (5).

## Discussion

Renal metastases in any primary tumor are uncommon in clinical practice. They are detected clinically at a lower rate than the prevalence based on autopsy data, which is estimated to be between 2.4% and 12.6% of patients with metastatic tumors (3). Renal metastases are primarily seen in lung, breast, digestive tract (esophagus, stomach, and colon), and melanoma (6). In one database of 365,099 autopsies and 835,959 metastatic organs, the major primary sites (range of proportions) of genitourinary organ metastases were the respiratory tract (5.6–34.0%), stomach (4.7–27.0%), hematologic site (0.9–24.9%), lymphoid (2.4–22.2%), bladder (0.8–20.0%), prostate (0.7–14.1%), rectum (2.0–11.7%), and pancreas (2.6–11.0%) (7). Imaging aids in diagnosis include CT, MRI, and ultrasound. The treatment should be individualized and consider the origin of the primary tumor, burden of disease, patient performance status, and other organs involved (8).

### Resection and Metastasectomy in non-RCC disease

Metastasectomy and resection of nonrenal cell carcinoma (RCC) histologies involving the kidney are pertinent topics for a urologist. One case report published in 2022 by Grandhi et al. describes nephrectomy, adrenalectomy, and regional lymphadenectomy of a 30 × 16 × 11 cm epithelioid angiomyolipoma (EAML), confirmed by fine-needle aspiration (FNA) to be malignant perivascular epithelioid cell neoplasm metastatic to the lung (9). This patient was managed by a team including thoracic surgery who confirmed metastatic EAML after VATS of lung nodule and medical oncology who initiated systemic temsirolimus, an mTOR-inhibitor, after restaging CT demonstrating new liver lesions. Another robotic nephroureterectomy and retroperitoneal lymph node dissection was performed by a urologic oncologist for a growing mass with neoplastic features on MRI. Pathology demonstrated the second known case of a hibernoma with the inflammatory variant and clinically, it was the first report of a hibernoma compressing the renal pelvis (10). The latter case report highlights that surgical excision is the diagnostic and treatment methodology of choice for renal masses that are clinically and radiologically indistinguishable from malignancy, even when renal mass biopsies are benign or nondiagnostic, as they can be about 14% of the time (8).

While ACC has a relatively slow clinical course, the long-term prognosis is poor due to high frequency of local recurrence and distant metastasis. Due to the rarity of the disease, few prospective clinical trials exist. And, due to the nature of the disease, few long-term outcome studies of greater than 20 years have been completed (11). In one single-institution study of 25 years, by Ishida et al., 50 patients were followed for 25 years. Over the observation period, in 35 (70.0%) of the 50 cases who underwent radical treatments, there was recurrence at locoregional sites (30%), distant/metastatic sites (36%), or both (4.0%) (11). In cases of locoregional recurrence, disease-specific survival was significantly improved in patients who underwent salvage treatment with surgery than with other modalities (p= 0.001), including CyberKnife, radiation, and particle beam therapy (11). However, in the patient cohort with metastases to the lungs, the most common site of metastases, the survival benefit of metastasectomy was greater than that of “best supportive therapy” (p=0.012) but not significantly better than other interventions including chemotherapy or radiation alone (11). While the optimal therapeutic approach remains to be identified in distant metastatic ACC, metastasectomy remains a viable option for select patient populations. The role of metastasectomy is most beneficial in a subset of patients who have potentially completely resectable metastatic tumors, appropriate performance status to undergo surgical intervention, and adequate affected-organ function.

### Importance of multidisciplinary treatment teams, communication, and patient counseling

This case report describes metastasectomy of ACC to the kidney managed by a multidisciplinary team. Patients with rare tumors such as ACC benefit from a highly collaborative approach. At our NCI-designated comprehensive cancer center, the proximity of subspecialty clinics and high frequency of tumor boards foster real-time collaboration and multidisciplinary care for new and established patients. In healthcare systems or across institutions where this may not be as easily attainable, obtaining outside records, pathology slides, and direct communication between team members is key. Intentional clinical documentation should highlight pertinent information with streamlined, concise documentation and focus on assessment and plan to clue multidisciplinary teams into each specialists’ assessment of severity and plan.

Proper and prompt patient counseling in complex or rare cases is rooted in effective multidisciplinary teams. To provide patients with consistent messaging and next-steps, teams will need to rely on communication via the medical record, email, clinics in physical proximity, and tumor boards. In addition, timely intervention can be facilitated by sending referrals to other specialists at time of visit or prior to intervention to account for scheduling delays and subsequent follow-up.

With regard to the urologist, patient counseling should also address operative treatment decisions including partial versus radical nephrectomy where applicable for anatomically complex tumors. Sparse, high-quality studies exist regarding the effectiveness of partial versus radical nephrectomy in the metastatic ACC population, including this case. The literature evidences that more complex tumors may be associated with more aggressive tumor biology and increased perioperative risk in patients undergoing cancer-related renal surgery (12, 13). A study by Kutikov et al. reviews the current literature for partial versus radical nephrectomy in large or anatomically complex, localized renal masses. They describe several validated scoring systems that quantify the complexity of renal tumors to facilitate decision-making, based on factors including tumor size, endophytic versus exophytic location, proximity to collecting system, hilar vessels, and degree of contact with parenchyma (14). Pre- and intraoperatively, the patient’s baseline health characteristics and anatomy of the tumor are factored into the decision to attempt a more complex partial nephrectomy to prioritize nephron-sparing status with potential for increased perioperative and oncologic risk versus proceeding with a total nephrectomy, sacrificing some normal parenchyma to ensure negative margins and a faster convalescence to receive systemic therapy. In this case, the tumor’s involvement in the renal pelvis, tumor staging with metastasis to the lung, and the patient’s normal glomerular filtration rate gave preference for radical nephrectomy with expedited time to systemic therapy.

## Conclusions

This is a case of recurrent ACC metastatic to the kidney managed by robotic radical nephrectomy and discussion of future adjuvant systemic therapy administered by a multidisciplinary treatment team at an NCI-designated comprehensive cancer center. Multidisciplinary communication, proper patient counseling, and examination of risk–benefit ratios should be considered in anatomically complex renal tumors. Counseling should include discussion of partial nephrectomy with prioritization of nephron-sparing with potential for increased perioperative risk versus radical nephrectomy to ensure negative margins and expedite timeline to systemic therapy.
